# Confirmatory prediction-driven RCTs in comparative effectiveness settings for cancer treatment

**DOI:** 10.1038/s41416-023-02144-x

**Published:** 2023-01-23

**Authors:** Adam Brand, Michael C. Sachs, Arvid Sjölander, Erin E. Gabriel

**Affiliations:** 1 Department of Medical Epidemiology and Biostatistics, Karolinska Institutet, Solna, Sweden; 2 Section of Biostatistics, Department of Public Health, University of Copenhagen, Copenhagen, Denmark

**Keywords:** Randomized controlled trials, Predictive markers

## Abstract

**Background:**

Medical advances in the treatment of cancer have allowed the development of multiple approved treatments and prognostic and predictive biomarkers for many types of cancer. Identifying improved treatment strategies among approved treatment options, the study of which is termed comparative effectiveness, using predictive biomarkers is becoming more common. RCTs that incorporate predictive biomarkers into the study design, called prediction-driven RCTs, are needed to rigorously evaluate these treatment strategies. Although researched extensively in the experimental treatment setting, literature is lacking in providing guidance about prediction-driven RCTs in the comparative effectiveness setting.

**Methods:**

Realistic simulations with time-to-event endpoints are used to compare contrasts of clinical utility and provide examples of simulated prediction-driven RCTs in the comparative effectiveness setting.

**Results:**

Our proposed contrast for clinical utility accurately estimates the true clinical utility in the comparative effectiveness setting while in some scenarios, the contrast used in current literature does not.

**Discussion:**

It is important to properly define contrasts of interest according to the treatment setting. Realistic simulations should be used to choose and evaluate the RCT design(s) able to directly estimate that contrast. In the comparative effectiveness setting, our proposed contrast for clinical utility should be used.

## Introduction

Advances in cancer treatment have allowed the development of multiple approved treatments for many types of cancer. Some of these treatments target-specific sub-types is defined by a biological mechanism. Identifying the optimal treatment for patients from multiple approved options is complex; the study of which is termed comparative effectiveness. Biomarker signatures, comprising one or more biomarker measurements, are used to determine the biological targets for specific treatments and/or to identify those patients expected to benefit from them. The use of these predictive biomarkers in cancer treatment is commonplace [1–10]. All treatments, targeted or otherwise, should provide compelling evidence of benefit compared to other approved treatment options through confirmatory randomised controlled trials (RCTs). RCTs designed specifically to evaluate treatments incorporating predictive biomarkers, or prediction-driven RCTs, are essential to future drug development and prescription [11]. Hu and Dignam provide an overview of the key concepts of prediction-driven RCTs [9].

Prediction-driven RCTs can be used to refine patient populations and identify superior treatment strategies when new biomarker signatures and treatments become available. NCI-MATCH is a high-profile platform trial that evaluates biomarker-directed treatment strategies, also referred to as prediction-driven decision rules, for underexplored cancer types [12]. ProBio is a platform RCT to identify new biomarker-directed treatment strategies that improve patient outcomes in metastatic castrate-resistant prostate cancer, currently comparing only among approved treatments [13]. The SHIVA trial evaluates molecular profiling to direct treatment of metastatic solid tumours [14]. Renfro et al. provide a review of prediction-driven RCTs along with additional examples of such trials [8]. The focus of this paper is the confirmatory comparative effectiveness setting, so we will limit focus of prediction-driven RCTs to trial designs amenable to frequentist analyses that can reliably control type 1 error. The prediction-driven RCT designs relevant to this setting fall into three categories: enrichment, biomarker-stratified and biomarker-strategy [8]. Although compared and evaluated extensively in the experimental treatment setting, these designs have not, to our knowledge, been evaluated and compared in a comparative effectiveness setting, which requires special consideration of clinical utility.

Clinical utility of a biomarker signature, defined as the improvement in patient outcome from having knowledge of the biomarker, is as important or more important than clinical validity, defined as the ability of a biomarker to accurately predict the effect of treatment; however, clinical utility is often overlooked [15]. A biomarker signature can have high clinical validity while also having little to no clinical utility. For example, consider a patient population in which a biomarker-signature accurately predicts which subgroup benefits most from which treatment, for example, high-risk patients benefit from aggressive treatment while low-risk patients benefit from milder treatment. If a physician can also classify patients accurately based on information routinely collected outside of the biomarker signature, then it has no clinical utility, because knowledge of the biomarker does not improve patient outcomes over the standard of care. Note that a biomarker cannot have clinical utility without clinical validity, because knowledge of a biomarker cannot improve outcomes if it cannot predict the outcome of treatment. Biomarker signatures can be costly and invasive for patients and should be avoided if they do not lead to improved outcomes, so the clinical utility of a biomarker signature should be rigorously evaluated before adopting into standard practice [15].

Evaluating clinical utility in RCTs depends heavily on the treatment setting. Consider for example the experimental treatment setting. What does it mean to be treated without knowledge of the biomarker in an experimental treatment setting? Even without knowledge of the biomarker the new treatment may improve outcomes. Authors who have evaluated the performance of RCT designs to estimate clinical utility in the experimental setting, such as Shih and Lin [16, 17] and Sargent and Allegra [18], have defined standard of care in the experimental setting as a randomised mix of experimental treatment and existing treatment. Comparing this standard of care to the biomarker-directed arm, one can test the global null that the biomarker-directed is the same as undirected treatment assignment. This definition of standard of care may be useful during the developmental stage in the experimental setting. In the comparative effectiveness setting, which is the focus of this paper, the treatment the patient would normally receive without knowledge of the biomarker status, as directed by a physician, is a more relevant definition of standard of care. We will refer to this definition of standard of care as physician’s choice throughout, and use it in our definition of clinical utility. We formally define clinical utility in section “Prediction-driven RCT designs and contrasts of interest”.

Evaluating clinical validity may be less valuable than evaluating clinical utility in the comparative effectiveness setting, but it can still be useful. As stated above, when a biomarker-signature is either costly or invasive, it should be evaluated for clinical utility, and clinical utility implies clinical validity. Therefore, evaluating both may not be efficient. However, when there are multiple biomarker-directed treatment strategies, as in ProBio [13], evaluating clinical validity, which typically requires smaller sample sizes [16–18], among the treatment strategy options before evaluating clinical utility in the best-performing treatment strategies may be a more efficient use of resources. Thus, RCTs specifically designed to detect clinical validity, or differential treatment effect between biomarker-defined subgroups, may also be useful in the comparative effectiveness setting.

In prediction-driven RCTs, it is common to evaluate the treatment effect for a particular subgroup. This is typically done in the experimental setting when it is thought that only one subgroup will benefit from a, frequently targeted, treatment. This may also be of value in the comparative effectiveness setting, for example, if a new biomarker is developed that is thought to identify a subgroup from a population that may benefit from a treatment previously shown to be inferior in the overall population. In this case, it may be unethical to randomise any patient not in the identified subgroup.

To our knowledge, there has not been a systematic comparison of confirmatory prediction-driven RCT designs in the comparative effectiveness setting. The prediction-driven RCT designs able to estimate clinical utility in the comparative effectiveness setting differ from those in the experimental setting, because the definition of standard of care is different. While it is still a question to us what the appropriate definition of standard of care is in the experimental setting, we propose a definition in the comparative effectiveness setting that provides easily interpretable results.

The primary aims of this paper are to define and motivate the use of our proposed contrast for clinical utility in the comparative effectiveness setting for cancer treatment research. We distinguish it from the other contrasts for measuring clinical utility that have been proposed in the experimental setting both theoretically and in a simulation study. We define other statistical contrasts of interest in the comparative effectiveness setting and demonstrate the prediction-driven RCT designs that can identify estimands for each contrast under minimal assumptions. For each estimand, we describe common/useful estimation options for practitioners considering such contrasts and trials in comparative effectiveness cancer treatment research. Finally, we provide step-by-step examples for simulating realistic RCTs and guidance on designing such trials using simulation.

### Prediction-driven RCT designs and contrasts of interest

Assume there are two approved treatment options, $X \in \left[ {A,B} \right]$, and that there is a biomarker signature that classifies the patient population for a specific cancer into two groups, positives (*M* = 1) and negatives (*M* = 0). Also assume that the proposed biomarker-directed treatment strategy is to treat all positive patients with treatment *B* and all negative patients with treatment *A*. Let *T* be the event time, or time to death (progression or failure), of a patient, possibly right-censored. Using counterfactual notation common in causal inference [19], let *T*_*B*_ be the counterfactual outcome of a patient had they been assigned to treatment *B* and likewise for *T*_*A*_. For a patient who is factually assigned to *B*, the factual outcome *T* equals the counterfactual outcome *T*_*B*_, whereas for a patient who is factually assigned to *A*, the factual outcome *T* equals the counterfactual outcome *T*_*A*_. Also, let $T_{{{{{{{{\mathrm{biomarker - directed}}}}}}}}}$ be the counterfactual outcome of a patient had they been assigned treatment according to the biomarker-directed strategy, that is, treating biomarker-positive patients with *B* and negative patients with *A*. Finally, let $T_{{{{{{{{\mathrm{physician - directed}}}}}}}}}$ be the counterfactual outcome of a patient had they been assigned treatment according to a physician’s prescribed treatment, choosing from either *A* or *B* without knowledge of biomarker status. The outcome *T* often represents a potentially right-censored time-to-event variable, but the contrasts defined below extend to any outcome. Let *g*(·) denote a summary statistic available for the outcome, *T*, such as the hazard or restricted mean survival time. Also assume that, as expected in an RCT for cancer treatment, there is no interference between patients. Then, the three contrasts of interest that we consider can be represented as below, with an absolute and relative difference presented for each.

### Treatment effect for a subgroup

$$g\left( {T_B|M = m} \right) - g\left( {T_A|M = m} \right)$$

or

$$\frac{{g\left( {T_B|M = m} \right)}}{{g\left( {T_A|M = m} \right)}}$$

### Clinical validity (differential treatment effect between subgroups)

$$\left[ {g\left( {T_B|M = 1} \right) - g\left( {T_A|M = 1} \right)} \right] - \left[ {g\left( {T_B|M = 0} \right) - g\left( {T_A|M = 0} \right)} \right]$$

or

$$\frac{{g\left( {T_B|M = 1} \right)}}{{g\left( {T_A|M = 1} \right)}}/\frac{{g\left( {T_B|M = 0} \right)}}{{g\left( {T_A|M = 0} \right)}} = \frac{{g\left( {T_B|M = 1} \right) \ast g\left( {T_A|M = 0} \right)}}{{g\left( {T_A|M = 1} \right) \ast g\left( {T_B|M = 0} \right)}}$$

### Clinical utility (proposed for comparative effectiveness)

$$g\left( {T_{{{{{{{{\mathrm{biomarker - directed}}}}}}}}}} \right) - g\left( {T_{{{{{{{{\mathrm{physician - directed}}}}}}}}}} \right)$$

or

$$\frac{{g\left( {T_{{{{{{{{\mathrm{biomarker - directed}}}}}}}}}} \right)}}{{g\left( {T_{{{{{{{{\mathrm{physician - directed}}}}}}}}}} \right)}}$$

**Fig. 1:**
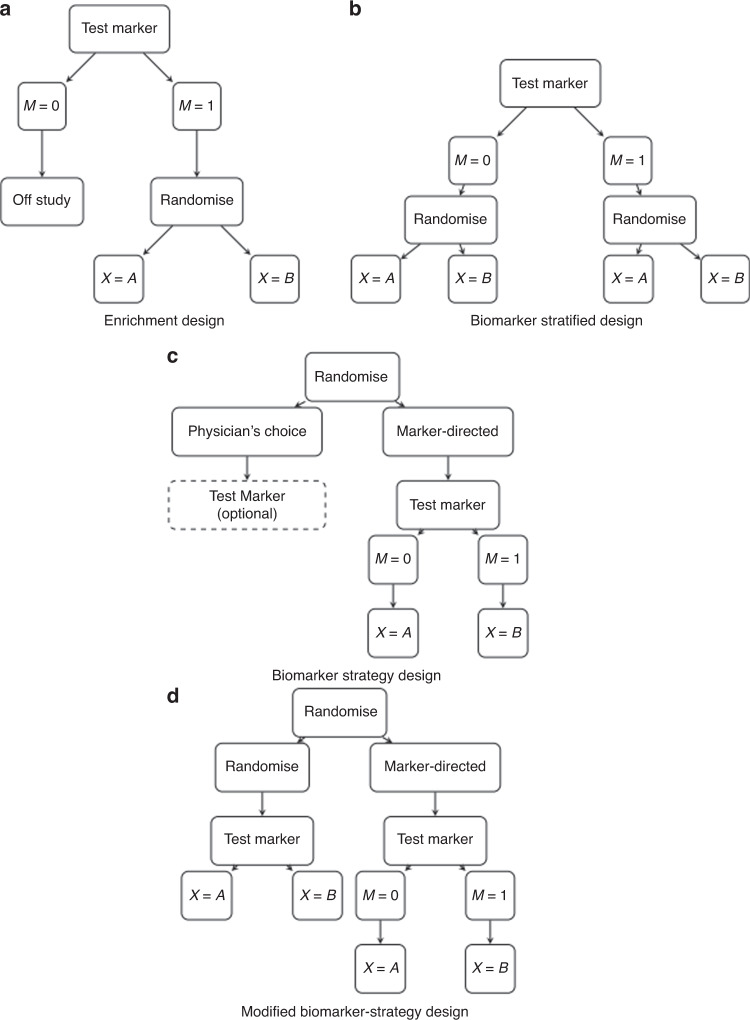
Prediction-driven RCT designs. The enrichment design, biomarker stratified design, biomarker strategy design and modified biomarker strategy design are depicted in panels (**a**), (**b**), (**c**) and (**d**), respectively. *M* denotes biomarker status and *X* denotes treatment assignment.

Figure 1 presents four prediction-driven RCT designs common to the literature of prediction-driven RCT designs [8, 18]: the enrichment design (a), the biomarker-stratified design (b), the biomarker-strategy design (c), and the modified biomarker-strategy design (d). Table 1 summarises which prediction-driven RCT designs can directly estimate which of the three contrasts of interest in this comparative effectiveness setting. The following subsections detail the ability of each design to estimate these contrasts.

**Table 1:** Identifiable contrasts when comparing approved treatments (A versus B) among biomarker-defined subgroups.

	Treatment effect in subgroup	Differential treatment effect	Clinical utility
Enrichment	X		
Biomarker-stratified	X	X	
Biomarker-strategy			X
Modified biomarker-strategy (biomarker testing in randomised arm)	X	X	
Modified biomarker-strategy (no testing in randomised arm)			

Note that in the comparative effectiveness setting, we define clinical utility as a biomarker-directed treatment strategy versus what a physician would prescribe without knowledge of the biomarker (physician’s choice). As discussed in the introduction this differs from the definition used in current literature on prediction-driven trials and alters the types of RCT designs that are able to estimate it. The contrast used in the experimental setting by Shih and Lin [17], for example, is as below where $T_{{{{{{{{\mathrm{randomized}}}}}}}}}$ is the counterfactual outcome for a patient whose treatment assignment was randomised.

### Clinical utility (experimental)

$$g\left( {T_{{{{{{{{\mathrm{biomarker - directed}}}}}}}}}} \right) - g(T_{{{{{{{{\mathrm{randomized}}}}}}}}})$$

or

$$\frac{{g\left( {T_{{{{{{{{\mathrm{biomarker - directed}}}}}}}}}} \right)}}{{g(T_{{{{{{{{\mathrm{randomized}}}}}}}}})}}$$

Specifically, while the biomarker-stratified and modified biomarker-stratified designs are able to estimate the experimental contrast for clinical utility, they are not able to estimate the proposed comparative effectiveness contrast above, as we discuss in the following subsections.

### Enrichment design

In an enrichment design, randomisation makes ignorable treatment assignment a plausible assumption. Thus, the following equalities hold:

1 $$g\left( {T_B|M = 1} \right) = g\left( {T|X = B,M = 1} \right)$$

2 $$g\left( {T_A|M = 1} \right) = g\left( {T|X = A,M = 1} \right)$$

Therefore, the contrast for treatment effect in the positive subgroup can be estimated by:

$$g\left( {T|X = B,M = 1} \right) - g\left( {T|X = A,M = 1} \right)$$

or

$$\frac{{g\left( {T|X = B,M = 1} \right)}}{{g\left( {T|X = A,M = 1} \right)}}$$

As biomarker-negative patients are not enroled in this design, the enrichment design cannot estimate the treatment effect in the negative subgroup without assuming a treatment effect distribution in the unobserved subgroup. Therefore, the enrichment design cannot estimate differential treatment effect nor clinical utility without that strong assumption.

### Biomarker-stratified design

The biomarker-stratified design can be viewed as two enrichment designs running in parallel, as randomisation is conducted within each of the subgroups. Therefore, (1) and (2) continue to hold while (3) and (4) are similarly true.

3 $$g\left( {T_B|M = 0} \right) = g\left( {T|X = B,M = 0} \right)$$

4 $$g\left( {T_A|M = 0} \right) = g\left( {T|X = A,M = 0} \right)$$

These equalities allow for estimating the treatment effect for each subgroup as in section “Enrichment design” and the differential treatment effect as below.

$$\left[ {g\left( {T|X = B,M = 1} \right) - g\left( {T|X = A,M = 1} \right)} \right] \\ - \left[ {g\left( {T|X = B,M = 0} \right) - g\left( {T|X = A,M = 0} \right)} \right]$$

or

$$\frac{{g\left( {T|X = B,M = 1} \right)}}{{g\left( {T|X = A,M = 1} \right)}}/\frac{{g\left( {T|X = B,M = 0} \right)}}{{g\left( {T|X = A,M = 0} \right)}} \\ = \frac{{g\left( {T|X = B,M = 1} \right) \ast g\left( {T|X = A,M = 0} \right)}}{{g\left( {T|X = A,M = 1} \right) \ast g\left( {T|X = B,M = 0} \right)}}$$

Unlike in the experimental setting referred to in Shih and Lin [16, 17] and Sargent and Allegra [18], the biomarker-stratified design cannot directly estimate clinical utility in the comparative effectiveness setting. Although randomisation allows estimating the summary statistic for the biomarker-directed arm, it cannot directly estimate the physician-directed arm, because a physician may assign treatment differently from the randomised assignment performed in the biomarker-stratified design.

### Biomarker-strategy design

The biomarker-strategy design, by randomising to a biomarker-directed arm versus a physician’s choice arm, directly evaluates the clinical utility of a biomarker signature. From randomisation it follows that,

$$g\left( {T_{{{{{{{{\mathrm{biomarker}}}}}} -{{{{{\mathrm{directed}}}}}}}}}} \right) = g\left( {T|{{{{{{{\mathrm{arm}}}}}}}} = {{{{{{{\mathrm{biomarker}}}}}} \!-\!{{{{{\mathrm{directed}}}}}}}}} \right),$$

and

$$g\left( {T_{{{{{{{{\mathrm{physician - directed}}}}}}}}}} \right) = g\left( {T|{{{{{{{\mathrm{arm}}}}}}}} = {{{{{{{\mathrm{physician}}}}}} \!-\!{{{{{\mathrm{directed}}}}}}}}} \right)$$

And so the contrast for clinical utility in the comparative effectiveness setting is

$$g\left( {T|{{{{{{{\mathrm{arm}}}}}}}} = {{{{{{{\mathrm{biomarker}}}}}} \!-\!{{{{{\mathrm{directed}}}}}}}}} \right) - g\left( {T|{{{{{{{\mathrm{arm}}}}}}}} = {{{{{{{\mathrm{physician}}}}}} \!-\!{{{{{\mathrm{directed}}}}}}}}} \right)$$

or

$$\frac{{g\left( {T|{{{{{{{\mathrm{arm}}}}}}}} = {{{{{{{\mathrm{biomarker}}}}}} \!-\!{{{{{\mathrm{directed}}}}}}}}} \right)}}{{g\left( {T|{{{{{{{\mathrm{arm}}}}}}}} = {{{{{{{\mathrm{physician}}}}}} \!-\!{{{{{\mathrm{directed}}}}}}}}} \right)}}$$

Note that because there is no randomisation to treatment *A* versus *B* within either subgroup, treatment effect within subgroups cannot be directly estimated with the biomarker-strategy design. Therefore, this design is not able to estimate treatment effect for either subgroup nor differential treatment effect without strong additional assumptions.

### Modified biomarker-strategy design

The modified biomarker-strategy design, proposed by Sargent and Allegra [18] and discussed in Shih and Lin [17], is a hybrid of the biomarker-stratified and biomarker-strategy designs. It compares a biomarker-directed treatment arm to a fully randomised arm, with or without stratification by marker status. If biomarker status is not obtained in the fully randomised arm, only (1) and (4) hold true, so neither treatment effect in a subgroup nor differential treatment effect can be estimated without assuming treatment effect distributions in (2) and (3). Also, there is no direct estimate available for the outcome in the physician’s choice arm, so clinical utility is also unestimable without strong additional assumptions.

If biomarker status is obtained in the randomised arm, then any modified biomarker-strategy design can be replicated with a biomarker-stratified design by altering the randomisation probabilities for each subgroup to match those in the modified biomarker-strategy design. Let *r*_*pos*1_ be the probability of a positive patient being randomised to treatment *B* in a biomarker-stratified design, *r*_strat_ be the probability of being randomised to the biomarker-directed treatment arm in the modified biomarker-strategy design, and *r*_pos2_ be the probability of a positive patient who was randomised to the randomised arm in the modified biomarker-strategy design being further randomised to treatment *B*. Then the probability of a positive patient assigned to treatment *B* in the modified biomarker-strategy design is $P\left( {M = 1,X = B|{{{{{{{\mathrm{modified}}}}}}}}\;{{{{{{{\mathrm{biomarker}}}}}}}}\;{{{{{{{\mathrm{strategy}}}}}}}}\;{{{{{{{\mathrm{design}}}}}}}}} \right) = r_{strat} + \left( {1 - r_{strat}} \right) \ast r_{pos2}$. Setting this probability equal to *r*_pos1_ ensures equal randomisation probabilities for both designs. Owing to randomisation, (1)–(4) all hold in both designs, so their ability to estimate the contrasts of interest are equal.

### Estimands and estimators

There are multiple ways to quantify differences in the distributions of censored time-to-event outcomes, and no option is superior for every setting. In this section, we discuss four common and/or useful estimands for differences in survival: logrank statistic (LR), hazard ratio (HR), difference in survival probability at a pre-specified time (SD), and absolute differences in restricted mean survival time (RMST) at a pre-specified time. Other estimands can certainly be appropriate.

LR is not appropriate for testing for a differential treatment effect nor can it directly estimate any of the contrasts in section “Prediction-driven RCT designs and contrasts of interest”. However, it is the most commonly used statistic for testing for differences in survival between two groups in confirmatory RCTs for cancer treatment. The *HR* is an estimand of the relative difference representations for each of the contrasts in section “Prediction-driven RCT designs and contrasts of interest”, while the SD and RMST are estimands of the absolute difference representations.

For estimation of SD and RMST, a pseudo-observation technique developed by Andersen et al. [20] is used. We chose this method of estimation due to its non-parametric modelling of the outcome and ease and flexibility of incorporating covariates, which is useful when estimating differential treatment effect and/or including baseline covariates predictive of outcome; other estimators are of course possible.

### Logrank statistic

The well-known logrank statistic is often used to test whether censored time-to-event distributions are different between two groups [21, 22]. Let *S*_1_ and *S*_2_ be survival distributions for groups 1 and 2, respectively. The null hypotheses corresponding to the contrasts in section “Prediction-driven RCT designs and contrasts of interest” are $H_0:S_{X = B,M = m} = S_{X = A,M = m}$ for the treatment effect in a single subgroup and $H_0:S_{{{{{{{{\mathrm{biomarker - directed}}}}}}}}} = S_{{{{{{{{\mathrm{physician - directed}}}}}}}}}$ for clinical utility of a biomarker signature.

LR does not provide a measure of the magnitude of survival differences. It can only test that survival distributions are equal. The logrank statistic is also not appropriate for testing for a differential treatment effect, because that test is of the null hypothesis that all four survival distributions are equal. If there is an equal, non-zero treatment effect in both subgroups, then the null hypothesis that the LR tests is false despite the absence of a differential effect.

### Hazard ratio

The hazard ratio (HR) is a common estimand of a magnitude of differences in survival using censored data that compares hazards, or the instantaneous risks of death (progression or failure), between two groups. Cox proportional hazards regression, proposed by Cox et al. [23], is used to estimate *HR* in our simulations below, which is valid for estimating a treatment effect under three conditions:

1. the hazard of death (progression or failure) is the same for censored and uncensored subjects, at all times *t*
2. the hazards in each group are proportional to the other hazards at all times *t*
3. both *T*_*A*_ and *T*_*B*_ are independent of *X*, as is the case in a confirmatory RCT for cancer treatment

One of the benefits of using the Cox model to estimate *HR* is that it allows for the adjustment of covariates by including them in the model. In this way, we can estimate the differential treatment effect as in (6). When estimating the contrasts in section “Prediction-driven RCT designs and contrasts of interest”, *g*(·) is the hazard, and the following Cox models will be fit,

5 $$\lambda \left( {t;X} \right) = \lambda _0\left( t \right)e^{\beta _0 \cdot I\left( {X = B} \right)}$$

6 $$\lambda \left( {t;X,M} \right) = \lambda _0\left( t \right)e^{\beta _0 \cdot I\left( {X = B} \right) + \beta _1 \cdot M + \beta _2 \cdot I\left( {X = B} \right) \cdot M}$$

7 $$\lambda \left( {t;ARM} \right) = \lambda _0\left( t \right)e^{\beta _0 \cdot I\left( {{{{{{{{\mathrm{biomarker - directed}}}}}}}}} \right)}$$

where *λ*_0_(*t*) represents the baseline hazard. Equation (5) estimates the treatment effect within a single subgroup as $e^{\beta _0}$, (6) estimates the differential treatment effect as $e^{\beta _2}$, and (7) estimates the clinical utility of the biomarker signature as $e^{\beta _0}$. The corresponding tests for statistical significance are based on $H_0:\beta _0 = 0$, $H_0:\beta _2 = 0$ and $H_0:\beta _0 = 0$, respectively.

The disadvantages of estimation via the hazard ratio are the reliance on the proportional hazards assumption and the complex interpretation [24, 25]. There are formal tests to indicate evidence of a departure from proportional hazards, and any procedure to test the assumption of proportional hazards and modify the analysis if needed should be carefully pre-specified in the trial study analysis plan prior to unblinding of treatment assignment. Hernan details the implications of deviations from proportional hazards on estimation and interpretation [26].

### Difference in survival probability at a pre-specified time

The difference in survival probabilities (SD) estimand is the difference of two groups’ probability of surviving past a specified time point, that is,

$$SD\left( t \right) = P\left( {T_1 \, > \, t} \right) - P\left( {T_2 \, > \, t} \right)$$

for groups 1 and 2.

SD can be estimated using a pseudo-observation technique developed by Andersen et al. [20], which uses non-parametric, Kaplan–Meier-based modelling of right-censored survival data while allowing for the adjustment of covariates [27]. Klein et al. applies this technique to the comparison of survival probabilities at fixed time points, and shows that it works well when incorporating covariates and when the proportional hazards assumption is violated [28]. Overgaard et al. presents the asymptotic theory of the pseudo-observation technique and proves that the estimating procedure used by Klein et al. is consistent under a condition of completely independent censoring, meaning that censoring is independent of event time, event type and covariates [29].

The pseudo-observation technique for censored time-to-event variables is as follows. Let *T*_*i*_, *i* from $1,...,n$, be independent and identically distributed time-to-event variables, and let *θ* be the expected value of some function of *T*_*i*_, that is $\theta = E\left[ {f\left( {T_i} \right)} \right]$ where *θ* may be multivariate. Also assume that there is an consistent estimator of *θ*, $\hat \theta$, and suppose there are measured covariates **Z**_*i*_. The *i*th pseudo-observation is defined by,

$$\hat \theta _i = n \cdot \hat \theta - \left( {n - 1} \right)\hat \theta ^{ - i}$$

where $\hat \theta ^{ - i}$ is the “leave-one-out” estimator for *θ*. Regressing on **Z** now corresponds to specifying the relationship between *θ*_*i*_ and **Z**_*i*_ using a generalised linear model with link function $\phi \left( \cdot \right)$,

$$\phi \left( {\theta _i} \right) = \beta ^T{{{{{{{\mathbf{Z}}}}}}}}_i$$

To estimate SD, one sets $f\left( {T_i} \right) = I\left( {T_i \, > \, t} \right)$, lets $\phi \left( \cdot \right)$ be the identity link function, and computes the pseudo-observations at a single time point, *t*. The pseudo-observations are then regressed according to the following models,

8 $$\theta _i\left( t \right) = \beta _0 + \beta _1 \cdot I\left( {X = B} \right)$$

9 $$\theta _i\left( t \right) = \beta _0 + \beta _1 \cdot I\left( {X = B} \right) + \beta _2 \cdot M + \beta _3 \cdot I\left( {X = B} \right) \cdot M$$

10 $$\theta _i\left( t \right) = \beta _0 + \beta _1 \cdot I\left( {{{{{{{{\mathrm{biomarker}}}}}} \!-\! {{{{{\mathrm{directed}}}}}}}}} \right)$$

where (8) estimates treatment effect for a subgroup as *β*_1_, (9) estimates differential treatment effect as *β*_3_, and (10) estimates clinical utility of the biomarker signature as *β*_1_. The corresponding tests for statistical significance are based on $H_0:\beta _1 = 0$, $H_0:\beta _3 = 0$ and $H_0:\beta _1 = 0$, respectively.

Although not dependent on the proportional hazards assumption, SD may be heavily dependent on the time point chosen to compare survival probabilities. When survival distributions cross, estimating SD at different times, even without bias, can provide qualitatively different results.

### Restricted mean survival time (RMST) at a pre-specified time

Restricted mean survival time (RMST), proposed by Irwin [30], is also estimated using the pseudo-observation technique, which was extended to estimate RMST by Andersen et al. [31]. RMST is defined as the average survival time up to time *t*, that is, $E\left[ {min\left( {T,t} \right)} \right]$. It can also be expressed as the area under a survival curve up to time *t*, that is,

$${{{{{\mathrm{RMST}}}}}} = {\int}_0^t {S\left( u \right)du}$$

and the estimate of RMST is,

$$\hat \theta = {\int}_0^t {\hat S\left( u \right)du}$$

To estimate RMST, one sets $f\left( {T_i} \right) = min\left( {T_i,t} \right)$, lets $\phi \left( \cdot \right)$ be the identity link, and computes the pseudo-observations at a single time point, *t*. The regression equations used to estimate the contrasts of interest in section “Prediction-driven RCT designs and contrasts of interest” are similar to (8)–(10). So then (8) estimates treatment effect for a single subgroup as *β*_1_, (9) estimates differential treatment effect as *β*_3_, and (10) estimates clinical utility of the biomarker signature as *β*_1_. The null hypotheses for testing for statistical significance are as in section “Difference in survival probability at a pre-specified time”.

This method of estimating RMST compares entire survival curves up to a time point while not relying on the proportional hazards assumption nor that survival curves do not cross. RMST may still be sensitive to the choice of *t*, because it ignores all information after *t*. However, it is not as sensitive as SD, because unlike SD, RMST incorporates all information in the survival function up until time *t*.

### Simulation

Simulations similar to those proposed by Rubenstein et al. [32] are used to closely simulate the conduct of actual prediction-driven, comparative effectiveness RCTs using time-to-event outcomes, providing estimates of relevant operating characteristics under assumed/estimated parameters. The code for the simulations is written in R and is publicly available at github.com/Adam-Brand/Prediction_Driven_Trials. The Supplement details the steps taken for the simulations in this paper as well as example simulations for each of the estimands of interest. These simulations can be used to design specific RCTs based on estimated/assumed inputs such as minimum clinically beneficial treatment effect and expected event rate.

To illustrate the potential for error in assessing clinical utility using the experimental estimand in the comparative effectiveness setting, we conducted a simulation with ideal physician’s choice of treatment. This means that the physician will always prescribe treatment according to the marker-directed treatment strategy and can occur if there are other readily-available patient data, which can accurately predict the biomarker status of the patient without obtaining biomarker status. As discussed in the introduction, this represents a scenario where the true clinical utility is zero, because knowledge of the biomarker cannot improve treatment outcomes.

Survival times are generated as independent draws from an exponential distribution, where median survival time is set separately for each of the four subgroups defined by biomarker status and treatment assignment, and survival times are independent of any other factors. Median survival for positive patients on treatment A, negative patients on treatment B and negative patients on treatment A is set to 9, 9, and 12 months, respectively. Median survival for positive patients on treatment B is set to 9, 12 and 21 months in different scenarios. Estimation is based on 1000 trials for each scenario, with varying effect sizes and proportion of biomarker positives.

## Results

Table 2 presents results comparing the experimental contrast for clinical utility to the proposed contrast for clinical utility in the comparative effectiveness setting. As discussed above, true clinical utility in this scenario is zero, so the true HR is 1 and the true RMST and SD are zero. As shown, using the experimental contrast for clinical utility drastically inflates the 0.05 type 1 error and distorts the true measures using any estimation method. Using the proposed contrast for clinical utility generally maintains the desired type 1 error under the null in this comparative effectiveness setting and estimates the true clinical utility accurately using any estimation method.

**Table 2:** Comparing contrasts of clinical utility over different estimands in the comparative effectiveness setting with ideal physician choice (no clinical utility).

Median (*T* | *M* = 1, *X* = *B*)	Marker pos.	LR reject		HR reject		HR mean		RMST reject		RMST mean		SD reject		SD mean	
		Exp.	Comp.	Exp.	Comp.	Exp.	Comp.	Exp.	Comp.	Exp.	Comp.	Exp.	Comp.	Exp.	Comp.
9	0.25	0.78	0.04	0.99	0.04	1.22	1.00	0.74	0.04	3.03	0.02	0.47	0.05	0.07	0.00
9	0.50	0.99	0.06	1.00	0.06	1.31	1.00	0.98	0.05	4.27	0.06	0.86	0.06	0.09	0.00
12	0.25	0.79	0.05	0.99	0.05	1.23	1.01	0.74	0.06	3.06	0.04	0.46	0.06	0.07	0.00
12	0.50	0.99	0.05	1.00	0.05	1.31	1.00	0.99	0.05	4.31	0.09	0.87	0.04	0.09	0.00
21	0.25	0.77	0.05	0.98	0.05	1.22	1.01	0.72	0.05	3.02	0.00	0.46	0.04	0.07	0.00
21	0.50	0.99	0.04	1.00	0.04	1.30	1.00	0.98	0.05	4.25	0.04	0.86	0.04	0.09	0.00

### Guidance for designing prediction-driven comparative effectiveness RCTs

It is important to define the target contrast and simulate using the design(s) able to directly identify that contrast when designing an RCT. Realistic simulations should be used based on estimated parameters, when possible, to achieve the desired operating characteristics, and the final design choice should be conservative with respect to those simulation results, that is, favour a larger sample size. Details of how to conduct a realistic simulation in this setting and examples of such simulations for each of the contrasts of interest are provided in the supplement. These simulations can be used to design specific RCTs based on estimated/assumed inputs such as minimum clinically beneficial treatment effect and expected event rate.

Previous literature has argued against the use of the biomarker-strategy design due to inferior efficiency, defined as larger sample size to achieve equal power [17, 18, 33]. However, the definition of clinical utility in these comparisons is questionable for the comparative effectiveness setting, comparing a biomarker-directed treatment arm to either single option treatment, that is, ignoring the treatment effect in one of the subgroups, or comparing to randomised treatment, which is not the standard of care in the comparative effectiveness setting. Defining clinical utility this way allows for estimation of clinical utility using the biomarker-stratified design, but as shown in Table 2, can produce results far from the truth. In the comparative effectiveness setting, the definition proposed in section “Prediction-driven RCT designs and contrasts of interest” should be used to provide a direct, interpretable estimate of clinical utility. The biomarker-strategy design is the only design in the literature able to directly estimate this definition of clinical utility.

We provide four useful options for quantifying the contrasts of interest in the comparative effectiveness setting for cancer research. Other options can be appropriate or even superior depending on the specific setting. Reasonable estimating options should be vetted through accurate simulations similar to those described in the supplement. Practitioners are encouraged to use simulations based as closely as possible to their setting to determine the best estimator in their setting given their contrast of interest.

## Discussion

We propose a definition and a set of estimands for quantification of clinical utility that are appropriate for the comparative effectiveness setting. We motivate the use of our proposed definition and estimands for clinical utility in this setting in comparison to an estimand and definition previously proposed and used in the experimental literature. We explain and demonstrate in simulations why these two concepts of clinical utility differ. We highlight that the RCT designs able to directly estimate estimands under this definition of clinical utility are not as previously reported in the experimental literature. We define some possible estimands for this contrast of interest in this setting, describe the RCT designs able to estimate them, and evaluate viable options for estimation.

We additionally consider other contrasts that may be of interest in the comparative effectiveness settings, suggesting estimands, estimators and RCTs designs that are useful for them. Using these illustrations and demonstrations of the contrasts, we provide guidance for the use of prediction-driven RCTs in the comparative effectiveness setting for cancer research. We also provide a guide to realistic trial simulation and guidance for designing such RCTs using simulations.

Although our simulations and discussion involve only two treatment options and two biomarker-defined subgroups, the designs and analyses can easily be extended to multiple treatment options and subgroups. Following the steps in the simulation guide in the supplement can provide estimated operating characteristics for trials with several treatment options and/or subgroups.

We call attention to the fact that previous definitions of clinical utility in the experimental RCT setting may lack interpretability/usefulness. Future research should explore the definition of clinical utility further in the experimental setting. Another potential area of future research is the estimation of the above contrasts in observational data; this would extend the work of Sachs et al. [15].

## Supplementary information

Supplement text

Figure S1

Figure S2

Figure S3

Table S1

Table S2

Table S3

## Data Availability

All code for the simulations, including the code to simulate the datasets, is publicly available at github.com/Adam-Brand/Prediction_Driven_Trials.
