# Supplementary material for: Confirmatory prediction-driven RCTs in comparative effectiveness settings for cancer treatment
Source: Br J Cancer. 2023 Jan 23;128(7):1278–85. doi: 10.1038/s41416-023-02144-x (PMC10050232; doi:10.1038/s41416-023-02144-x)
Supplement: Supplementary file 1 — Supplement text [file 41416_2023_2144_MOESM1_ESM.pdf]

Supplement for "Confirmatory Prediction-driven RCTs in Comparative Effectiveness Settings for Cancer Treatment"

1 Simulations

Simulations similar to those proposed by Rubenstein et al.[1] are used to closely simulate the conduct of actual prediction-driven, comparative effectiveness RCTs using time-to-event outcomes, providing estimates of relevant operating characteristics under assumed/estimated parameters. The simulations are written in R, the code for which is publicly available at `github.com/Adam-Brand/Prediction_Driven_Trials`. Supplement Section 1 details the steps taken for the simulations in this paper. Section 2 provides a general step-by-step procedure to simulate RCTs in any setting.

We simulated a trial with sequential enrollment as follows. Let L_i denote entry time for subject i, $i = 0, 1, ...$, counted from the start of the trial. We defined L_i iteratively as

$$\begin{aligned} L_0 &= 0 \\ L_i &= \sum_{i=0}^{i-1} L_i + W_i, \end{aligned}$$

where $W_1, W_2, ...$ are independent and

$$W_i \sim Exp(10).$$

Following the notation in the main text, let $M_i \in \{0, 1\}$ and $X_i \in \{0, 1\}$ be the biomarker and treatment for subject i, respectively, where we have for convenience coded treatment levels A and B as 0 and 1, respectively. We simulated M_i as

$$M_i \sim Ber(p).$$

The simulation of X_i depends on the design. For the enrichment design and the biomarker-stratified design, we simulated X_i as

$$X_i \sim Ber(p_x).$$

For the biomarker strategy design, let S_i be the indicator of being randomized to the biomarker-directed arm for subject i. For this design we simulated S_i and X_i as

$$\begin{aligned} S_i &\sim Ber(p_{bd}) \\ X_i &\sim Ber(1 - p_{pc}) \text{ if } S_i = 0, M = 0_i \\ X_i &\sim Ber(p_{pc}) \text{ if } S_i = 0, M_i = 1 \\ X_i &= M_i \text{ if } S_i = 1 \end{aligned}$$

Under this model, p_{pc} is the probability that the physician's choice agrees with the biomarker-directed strategy; we assumed that this probability does not depend on the true value of the biomarker. Let T_i and Δ_i be the survival time counted from time at entry and the indicator of loss-to-follow-up for subject i, respectively. We simulated T_i and Δ_i as

$$\begin{aligned} T_i &\sim Exp(\lambda_{XM}) \\ \Delta_i &\sim Ber(l) \end{aligned}$$

Using T_i and Δ_i, we simulated the time Z_i that subject i would be followed, counted from time at entry, if there were no administrative end of follow up:

$$\begin{aligned} Z_i &= T_i \text{ if } \Delta_i = 0 \\ Z_i &\sim Unif(0.001, T_i) \text{ if } \Delta_i = 1 \end{aligned}$$

For each scenario, we generated enough vectors $(L_i, M_i, X_i, T_i, \Delta_i, Z_i)$ sequentially from the model above (also including S_i in the vector for the biomarker-directed design) to achieve a pre-specified number K of outcome events while accruing patients up until K events were observed. For each patient in our sample where $\Delta_i = 0$ we defined the event study time, V_i, as

$$V_i = L_i + Z_i \text{ if } \Delta_i = 0$$

We then ordered the study events times, V_i, in increasing order. Let O_q be the qth order statistic of the increasingly ordered V_i's. We define the study administrative end of follow up, T_{EOF}, as the Kth order statistic of O:

$$T_{EOF} = O_K$$

Patients whose $L_i \geq T_{EOF}$ were then removed from the generated vectors. For patients whose $L_i < T_{EOF}$, we computed the observed follow-up time T_i^* and observed event indicator $\Delta_i^* = 0$ for subject i as

$$\begin{aligned} T_i^* &= \min(Z_i, T_{EOF} - L_i) \\ \Delta_i^* &= \Delta_i \text{ if } V_i \leq T_{EOF} \\ \Delta_i^* &= 1 \text{ if } V_i > T_{EOF} \end{aligned}$$

Thus, the observed data for subject i consists of $(L_i, M_i, X_i, \Delta_i^*, T_i^*)$ for the enrichment design and stratified-biomarker design, and $(L_i, M_i, X_i, \Delta_i^*, T_i^*, S_i)$ for the biomarker directed design, where the subject has the outcome event at time T_i^* if $\Delta_i^* = 0$, and is otherwise censored at T_i^*.

We used parameter values $p \in \{0.25, 0.5\}$, $l = 0.02$, $p_x = 0.5$, $p_{bd} = 0.5$, $p_{pc} \in \{0.25, 0.5, 0.75, 1\}$. The rate parameter λ_{XM} was determined based on likely median survival times for cancer treatment patients. Let Med_{XM} be the median survival for patients assigned to treatment X with biomarker status M. $Med_{XM} \in \{9, 12, 15, 21\}$ and

$$\lambda_{XM} = ln(2)/Med_{XM}$$

Generating survival times as above ensures proportional hazards between subgroups, but generally violates proportional hazards when estimating clinical utility. A minimum clinically beneficial treatment effect is defined as an increase in median survival from 9 months to 12 months.

Estimation of treatment effect in a single subgroup is simulated using the enrichment design in 3 scenarios. In all scenarios, $Med_{A1} = 9$. In the other scenarios, Med_{B1} is one of 9, 12 or 21; the latter two scenarios reflecting increasing effect sizes while the first scenario is used to estimate type 1 error. We generated 1000 samples of size 3000 at each of the event sizes from 50 to 800, counting by 50 patients, in the latter two scenarios. The number of patients accrued is the mean over the 1000 trials of the number of patients whose $L_i < T_{EOF}$. Trial duration is the mean T_{EOF} over those trials. The power to detect a treatment difference is the proportion of trials to reject the null hypothesis of equal treatment effect based on a two-sided test of size 0.05 for each of the inference methods. The coverage for each method is the proportion of trials where the 95% confidence interval includes the true parameter. Type 1 error was calculated based on 1000 samples of size 3000 at the event size that produced roughly 90% power at each effect size; those event sizes were 550 when $Med_{B1} = 12$ and 150 when $Med_{B1} = 21$. Type 1 error was computed as the proportion of trials to reject the null hypothesis of equal treatment effect when both $Med_{A1} = 9$ and $Med_{B1} = 9$.

Estimation of differential treatment effect is simulated using the biomarker stratified design in 7 scenarios. Six scenarios with a non-zero differential treatment effect were simulated where $Med_{A1} = 9$, $Med_{B0} = 9$ and $Med_{A0} = 12$. The scenarios differ by a different combination of $M \in \{0.25, 0.50\}$ and $Med_{B1} \in \{9, 12, 15\}$. For those 6 scenarios, we generated 1000 samples of size 4000 at each of the event sizes from 250 to 1000, counting by 50 patients, and we generated 1000 samples of size 6000 at each of the event sizes from 1050 to 2000, counting by 50 patients. Summary measures were calculated as above, but with the null hypothesis that there was no differential treatment effect between biomarker-defined subgroups. Type 1 error was calculated based on 1000 samples of size 6000 at the event size that produced roughly 90% power for each scenario. Type 1 error was computed as the proportion of trials to reject the null hypothesis of no differential treatment effect when there was an equal, 3-month treatment effect size in each subgroup.

Estimation of clinical utility is simulated using the biomarker strategy design in 9 scenarios with a non-zero effect size. Each scenario represents a different combination of $p_{pc} \in \{0.25, 0.5, 1\}$ and $Med_{B1} \in \{9, 12, 21\}$. We generated 1000 samples of size 8000 for each scenario for event sizes from 500 to 3000, counting by 250 patients. Summary measures were calculated as above, but with the null hypothesis that there was no clinical utility of the biomarker, that is, the true summary measures were equal in the biomarker-directed arm and physician's choice arm. Type 1 error was calculated at event sizes that produced approximately 90% power in 6 of these scenarios (the omitted scenarios did not reach 90% power at the maximum event sizes explored) with $p_{pc} = 1$.

Logrank p-values, differences in expected number of events for contrasts of two groups, and median survival are calculated using the `survival` package. Cox models for estimating hazard ratios are fit using the `coxph` package. Pseudo-observations, used for estimating SD and $RMST$, are computed in R using the `pseudo` package. GLM models are fit using the `geese` package. For SD and $RMST$, approximate jackknife variance estimates from Yan and Fine[2] are used following the recommendation by Andersen et al.[3].

In all scenarios, the time point specified for SD is the median survival time of the least surviving subgroup. We found that choosing a later time point resulted in high variance of the estimate due to the lower number at risk at such time points. Choosing an earlier time point produced lower variance, but also lower power. For $RMST$, the maximum follow-up time was specified. We define the maximum follow-up time as the overall maximum observed time if the maximum observed time in the group with lower maximum observed time was an event. If the maximum observed time in the group with lower maximum observed time was censored, then that censored time is the maximum follow-up time. This definition ensures that there is no extrapolation of a group's survival curve beyond a last, censored observation time while also leveraging the

most amount of information. We found that pre-specifying an earlier follow-up time for $RMST$ was not as powerful in detecting treatment differences. Coverage probabilities are calculated as the proportion of trials where the 95% confidence interval of the estimate includes the true value.

1.1 Results

Figure S1 presents power curves for identifying treatment effect in a single subgroup with sample sizes ranging from 50 to 800 by increments of 50 patients. Figure S2 presents power curves for identifying differential treatment effect. Sample sizes range from 250 to 2000 when median survival in the biomarker positive group is set to 9 months and from 250 to 1000 when median survival in the biomarker positive group is set to 12 or 15 months. Figure S3 shows power curves for the biomarker strategy design identifying clinical utility with sample sizes ranging from 500 to 3000 by increments of 250 patients. In all figures, the x-axis, Events, is the number of observed events, or sample size, not the total number of patients accrued. Tables S1, S2 and S3 present the other operating characteristics for each estimand and scenario, presenting results for sample sizes achieving approximately 90% power. Note from Figure S3 that three scenarios do not obtain 90% power even at sample sizes up to 3000. These scenarios are not shown in Table S3. The operating characteristics shown in Tables S1-S3 include the average number of patients accrued, duration of the trial, type 1 error rates, and coverage probabilities for each estimand, except LR.

In general, HR and $RMST$ perform similarly in all operating characteristics. SD has lower power than the other estimands, as might be expected given it only uses information at a single time point whereas LR HR and $RMST$ use information from the whole survival curves. HR has poor coverage probability as the effect size and sample size increase. Reasons for this are discussed in the next Section. Type 1 error rate is generally controlled at the nominal level except when estimating differential treatment effect when the difference in treatment effect was low.

We note that in our simulations, the HR coverage probability of the true hazard ratio for clinical utility drastically decreases with increased sample size and proportion of physician-directed treatment equalling biomarker-directed treatment. This is because the survival distributions are simulated as a mixture of exponential distributions defined by both biomarker status and treatment assignment, which violates the proportional hazards assumption when patients are pooled into the biomarker-directed versus physician's choice arm. At the end of a trial, the Cox model estimating HR uses all information available, and the corresponding true hazard ratio can be defined as the ratio of cumulative hazards up until the maximum follow-up time. As the sample size increases, the expected value of the maximum follow-up time also increases. With increasing follow-up time, the departure from proportional hazards increases. The loss in coverage probability is not due to unstable estimates of HR, but rather that the true HR changes over time, leading to loss in coverage probability. In practice it is possible that a scenario occurs where proportional hazards holds true (or approximately so) within the clinical utility contrast, and this should be explored through simulations using estimated, if possible, or assumed survival distributions before choosing an analysis method.

2 Guide for RCT Simulations

1. **Identify the contrast(s) of interest and corresponding designs.** Determine which of the common contrasts of interest is most relevant to the question and the design(s) able to directly estimate it using Section 2 as a guide.

2. **Identify and estimate/assume relevant trial parameters.** Relevant trial parameters can be determined by the relevant operating characteristics of a trial design. The relevant operating characteristics we identified in this setting are: total number of subjects accrued, total time until completion of the trial, power of the trial to identify a pre-defined survival difference(s), type 1 error rate for declaring a statistically significant survival difference(s), and coverage probabilities of the estimates of the contrasts. Relevant trial parameters are those quantifiable trial parameters that have a direct effect on the relevant operating characteristics, and include, possibly among others: sample size (number of events), accrual rate, survival distribution for each subgroup defined by both biomarker status and treatment assignment, loss-to-follow-up rate, proportion of biomarker positives, and proportion of patients whose physician's choice of treatment agrees with the biomarker-directed treatment strategy.

   Estimate the trial parameters from recent observational data, if possible. If not possible, assume the range of likely values for the inestimable parameters. Whether estimated or assumed, the parameters should be conservative in that they should lead to larger necessary sample sizes than less conservative estimates/assumptions.

3. **Generate patient data.** First, identify the variables necessary for simulating an actual trial and for producing output of the relevant operating characteristics. The necessary variables we identified for this setting are: trial entry time, biomarker status, treatment assignment, physician's choice of treatment, event (death, progression or failure) time, a loss-to-follow-up indicator, loss-to-follow-up time, and an event indicator.

4. **Simulate N trials at different sample sizes.** Simulate a trial for each of the trial designs identified in step 2 (as detailed below), and repeat the process, starting with generating new patient data, N times. Do this for different sample sizes, starting with a low sample size and increasing until the desired operating characteristics are achieved.

   (a) Determine the treatment assignment for each patient based on the design and the treatment assignment variables.

   (b) Assign the event time as follows. If $LTFU = 1$, then assign loss-to-follow-up time as the event time. If $LTFU = 0$, assign the event time for treatment A or B according to treatment assignment.

   (c) Compute trial event time for each patient as the trial entry time plus the event time, and order the data by increasing trial event time.

   (d) Compute trial analysis time. For a sample of size n, set the trial analysis time equal to the trial event time for the n-th event.

   In calculating the n-th trial event time, include only those patients contributing to the contrast of interest and count only patients with event indicator equal to 1. For example, treatment effect for a single subgroup only considers one subgroup, so remove all patients from the excluded subgroup prior to calculation of trial completion time.

   In the simulations in this paper, subject recruitment continued until the trial analysis time, which reflects industry trials that attempt to minimize time until trial analysis. Alternatively, trials can be designed to minimize subject enrollment, in which case, simulations should stop recruitment when enough subjects have been recruited in order to provide the desired number of events by the desired time. As this is estimated, the number of subjects enrolled should be conservative, allowing for dropouts and slower than expected event rates.

(e) Remove all patients whose trial entry time is greater than the trial analysis time.

(f) Administratively censor all patients whose trial event time is greater than the trial analysis time by setting their event indicator to 0 and their event time to the trial analysis time minus their trial entry time.

(g) Using the completed trial data, estimate the contrasts of interest and output the results necessary to summarize the operating characteristics.

The following results are output in the simulations: number accrued, sample size (number of events), trial completion time, logrank p-value and difference in expected number of events for contrasts of two groups, median survival estimates for each subgroup, HR for all directly identifiable contrasts including confidence intervals and p-values, SD for all directly identifiable contrasts including confidence intervals and p-values, and $RMST$ for all directly identifiable contrasts including confidence intervals and p-values.

The number enrolled is simply the number of patients in the final trial data. Sample size (number of events) is pre-specified by the user, and trial analysis time is computed in Step 5d.

5. **Summarize trial operating characteristics.** After simulating N trials and outputting the results for each, summarize the relevant operating characteristics by calculating means and confidence intervals.

6. **Choose sample size; be conservative.** Compare the operating characteristics among different sample sizes, and select the minimum sample size with favorable results. Increasing sample size after the start of a trial can be complicated an have statistical implications. Therefore, it is best to be conservative when choosing a sample size.
