# Supplementary figures and images for: Confirmatory prediction-driven RCTs in comparative effectiveness settings for cancer treatment
Source: Br J Cancer. 2023 Jan 23;128(7):1278–85. doi: 10.1038/s41416-023-02144-x (PMC10050232; doi:10.1038/s41416-023-02144-x)

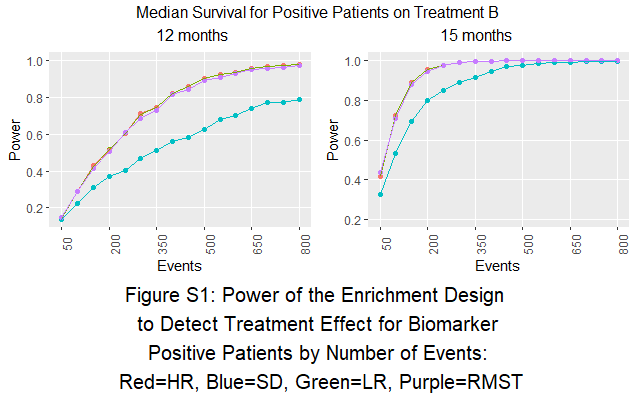

Supplement: Supplementary file 2 — Figure S1 [file 41416_2023_2144_MOESM2_ESM.png]

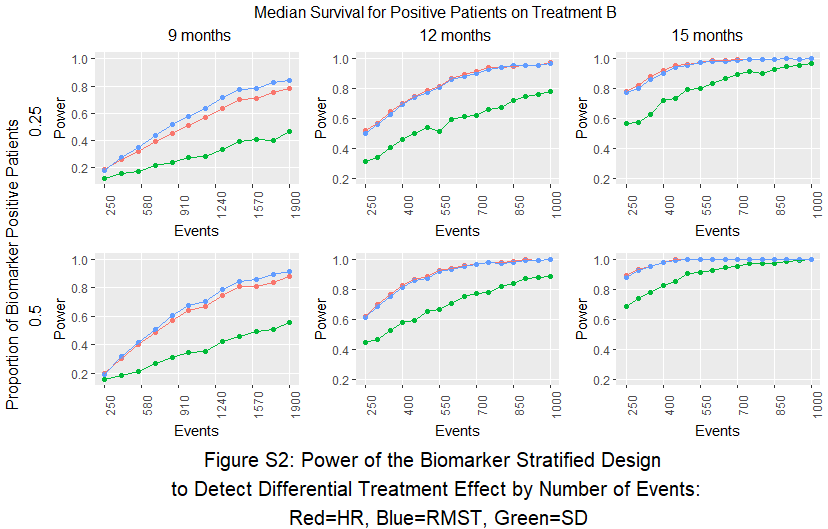

Supplement: Supplementary file 3 — Figure S2 [file 41416_2023_2144_MOESM3_ESM.png]

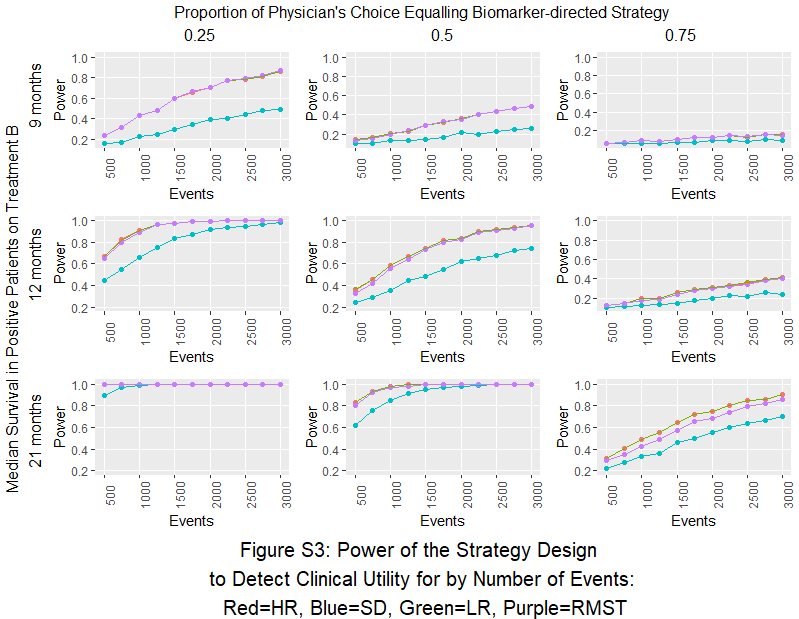

Supplement: Supplementary file 4 — Figure S3 [file 41416_2023_2144_MOESM4_ESM.png]
