# Supplementary material for: Confirmatory prediction-driven RCTs in comparative effectiveness settings for cancer treatment
Source: Br J Cancer. 2023 Jan 23;128(7):1278–85. doi: 10.1038/s41416-023-02144-x (PMC10050232; doi:10.1038/s41416-023-02144-x)
Supplement: Supplementary file 5 — Table S1 [file 41416_2023_2144_MOESM5_ESM.docx]

| **Table S1:** Operating Characteristics of the Enrichment Design with Approximately 90% Power to Detect Treatment Effect in Biomarker Positive Patients | | | | | | | | | | | | | | |
| --- | --- | --- | --- | --- | --- | --- | --- | --- | --- | --- | --- | --- | --- | --- |
|  |  |  |  |  |  |  |  |  |  |  |  |  |  |  |
| Median(T\|M=1,X=B | Events | Accrued | Duration (months) | LR power | LR T1E | HR power | HR coverage | HR T1E | SD power | SD coverage | SD T1E | RMST power | RMST coverage | RMST T1E |
| 12 | 550 | 698 | 77.2 | 0.93 | 0.051 | 0.93 | 0.95 | 0.051 | 0.68 | 0.96 | 0.048 | 0.91 | 0.95 | 0.049 |
| 15 | 150 | 281 | 31.0 | 0.89 | 0.039 | 0.89 | 0.96 | 0.039 | 0.69 | 0.95 | 0.042 | 0.88 | 0.96 | 0.040 |
| The first column is the median survival in biomarker positive patients who received treatment B. The median survival in positive patients who received treatment A, negative patients who received treatment B, and negative patients who received treatment A is 9, 9 , and 12, respectively (except in calculation of type 1 error). Events is the number of deaths observed during the trial. Accrued is the total patients accrued into the trial and Duration is the total trial duration in months. T1E denotes the type 1 error rate for each of the estimands and coverage denotes the proportion of 95\% confidence intervals contained the true parameter. | | | | | | | | | | | | | | |
